# Monogeneans on exotic Indian freshwater fish. 7. Results of a national study on ornamental fishes from 2019–2022

**DOI:** 10.1051/parasite/2025021

**Published:** 2025-05-12

**Authors:** Amit Tripathi, Chawan Matey, Kurt Buchmann, Christoph Hahn

**Affiliations:** 1 Department of Zoology, University of Lucknow Uttar Pradesh 226 007 India; 2 Department of Veterinary and Animal Sciences, University of Copenhagen Stigbøjlen 7 DK-1870 Frederiksberg C. Denmark; 3 Department of Biology, University of Graz Universitätsplatz 2 A-8010 Graz Austria

**Keywords:** Exotic ornamental fish, Monogenea, Photographic vouchers, Transboundary dissemination, 28S ribosomal RNA genes and ITS2 regions

## Abstract

This study reports the results of a nationwide parasitological survey that was conducted from 2019 to 2022 to investigate the potential introduction of monogenean parasites into India via the ornamental fish trade. A total of 619 individual exotic ornamental fish representing 27 teleost species from nine families were collected from the country’s major aquaria markets and examined for monogeneans. To identify monogeneans at the species level, we employed a morphometric analysis of sclerotised structures (haptoral and reproductive hard parts), as well as a molecular analysis of nuclear 28S rRNA and ITS2 regions. Indian conditions for importing exotic ornamental fish species require a pre-quarantine certificate, quarantine treatment, and post-quarantine follow-up. Despite these restrictions, 26 monogenean species from 12 known genera were detected and identified in 17 of the 27 fishes examined. *Dactylogyrus* was represented by a maximum of nine species, followed by *Gyrodactylus* with five. Cyprinidae was the most parasitised fish family (13 species), followed by Cichlidae (three species) and Helostomatidae, Poeciliidae, and Serrasalmidae (two species each). The majority of co-transported parasite species originated from Asia (65.38%, *n* = 17), followed by South America (23.07%, *n* = 6), North and Central America (7.69%, *n* = 2), and Africa (3.5%, *n* = 1). Three fish species were identified as the first host records for monogenean parasites: *Chindongo socolofi* for *Cichlidogyrus tilapiae* Paperna, 1960, *Metynnis hypsauchen* for *Mymarothecium* sp., and *Betta splendens* for *Heteronchocleidus* sp. In general, exotic populations had fewer parasite species than in their native distribution ranges.

## Abstract (fr)

Cette étude présente les résultats d’une enquête parasitologique nationale menée de 2019 à 2022 afin d’étudier l’introduction potentielle de monogènes parasites en Inde via le commerce de poissons d’ornement. Au total, 619 poissons d’ornement exotiques, représentant 27 espèces de téléostéens appartenant à neuf familles, ont été collectés sur les principaux marchés aquariophiles du pays et examinés à la recherche de Monogènes. Pour identifier les Monogènes à l’échelle de l’espèce, nous avons utilisé une analyse morphométrique des structures sclérifiées (parties dures du hapteur et de l’appareil reproducteur), ainsi qu’une analyse moléculaire des régions nucléaires de l’ARNr 28S et de l’ITS2. En Inde, les conditions d’importation des espèces de poissons d’ornement exotiques exigent un certificat de pré-quarantaine, un traitement de quarantaine et un suivi post-quarantaine. Malgré ces restrictions, 26 espèces de 12 genres connus de Monogènes ont été détectées et identifiées chez 17 des 27 poissons examinés. *Dactylogyrus* était représenté par un maximum de neuf espèces, suivi de *Gyrodactylus* avec cinq. Les Cyprinidae était la famille de poissons la plus parasitée (13 espèces), suivie des Cichlidae (trois espèces) et des Helostomatidae, Poeciliidae et Serrasalmidae (deux espèces chacune). La majorité des espèces de parasites co-transportés provenaient d’Asie (65,38 %, *n* = 17), suivie par l’Amérique du Sud (23,07 %, *n* = 6), l’Amérique du Nord et l’Amérique centrale (7,69 %, *n* = 2) et l’Afrique (3,5 %, *n* = 1). Trois espèces de poissons ont été identifiées comme premiers hôtes signalés pour des Monogènes : *Chindongo socolofi* pour *Cichlidogyrus tilapiae* Paperna, 1960, *Metynnis hypsauchen* pour *Mymarothecium* sp. et *Betta splendens* pour *Heteronchocleidus* sp. En général, les populations exotiques comptaient moins d’espèces de parasites que dans leurs aires de répartition d’origine.

## Introduction

The international trade in ornamental fish, invertebrates, and plants is gaining increasing importance globally. It connects all continents through significant transfer of this specialised form of pet organism between Asia, Europe, Africa, and the Americas [30, 69, 75, 109]. Economic prospects and lucrative markets have led to impressive expansion of trade in ornamentals, which has increased from around 1 billion USD in the 1990s [4, 21] to more than 6 billion USD recently [38]. However, the import of non-endemic organisms into new geographic areas has raised concerns due to the risk of introducing potentially invasive species into vulnerable habitats, where they may outcompete local and endemic species [31]. An additional area of concern has been raised by a series of reports documenting the concomitant import of parasites with their hosts [51, 108].

India, with its relatively modest yet vibrant aquaria market, is a global hub for freshwater ornamental fishes. In 2022, the country imported ornamental fish worth 2.44 million USD, accounting for 0.72% of all global imports [73]. Though the domestic market comprises both exotic and native species of ornamental fish, exotic species are prioritised [87]. More than 170 species of ornamental fish have been introduced into India via the aquarium trade, at least 18 of which now have permanent self-reproducing populations in natural waters, especially in peninsular India [99].

As this aquaria trade facilitates the translocation of almost all known exotic aquatic parasite groups [25, 32, 58, 107], it is a perfect gateway for the translocation of monogenean parasites [99]. Monogeneans parasitise the external surfaces of fish (gills, fins, and skin), involve only one host in their life cycle, and may occur in diverse habitats, ranging from freshwater via brackish water to marine water fishes [106]. These worms feed on the blood [43] and epithelial cells and mucus of fish [17], causing direct loss due to mortality, usually to younger fish and those in intensive culture or captive conditions [95].

Exotic monogeneans could pose a serious biological invasion challenge to the fish fauna of the destination environment because (in the absence of coevolution) the native fish species lack protective immunity against exotic parasites [85, 94]. The transfer of co-introduced monogeneans of ornamental fishes to the native wild fish and the latter’s subsequent deaths is not yet well understood. Nonetheless, the global co-introductions of exotic food fish and their monogenean parasites demonstrate the devastating impact exotic monogeneans can have on the importing aquaculture industry. One example is the translocation of *Gyrodactylus salaris* Malmberg, 1957 from native Baltic stocks of Atlantic salmon in Sweden to Norway in the 1970s. Due to innate immunity, Baltic stocks had developed a balanced coexistence with the parasite, limiting its spread. However, Norway’s East-Atlantic salmon stocks lacked natural resistance, which allowed *G. salaris* to rapidly spread on these fishes [26, 57] and reduce their population in more than 51 major Norwegian rivers [66]. This resulted in economic losses exceeding 500 million USD [7]. Likewise, due to decades of intercontinental eel trading, the gill monogeneans within the genus *Pseudodactylogyrus* Gusev, 1965 are now prevalent on European eel in Europe, on farms and in the wild [16]. Climate change may further aggravate the risk by providing more suitable habitats for both hosts and parasites [18].

India has a rich diversity of 1,044 freshwater fishes, with 196 endemic and 822 native fishes which are economically, ecologically, and culturally important [33]. Maintaining this diversity is an important challenge for future generations, which may be jeopardised by the introduction of exotic fish and their parasites via the aquaria trade. India, as a signatory to international conventions and organisations, particularly the Convention on Biological Diversity (CBD) and the WTO agreement on Sanitary and Phytosanitary Measures (SPS Agreement), which aim to conserve biological diversity by preventing invasive alien species, has enacted the “Sanitary protocol for import of ornamental fishes into India” to effectively control and manage the introduction of ornamental fish and associated diseases into the country. The guidelines implement a twin strategy of (1) developing an “indicative list” of 97 individual species cleared for import, and (2) imposing import procedures and requirements (pre-quarantine, quarantine, and post-quarantine). These guidelines prohibit the import of any species that is not on the indicative list.

In line with pre-quarantine actions, ornamental fish cannot be imported unless accompanied by a valid import permit issued by the Government of India and a pre-quarantine certificate from the exporting countries of the consignments. Under the quarantine actions, the imported species of fish are subjected to a mandatory quarantine protocol for 15 days (21 days for goldfish) in a quarantine facility. Per the post-quarantine follow-up guidelines, it is an offence to release fish or to allow fish to escape into the wild. In addition, the guidelines prohibit the direct sale of imported brood stocks in the domestic market, allowing only the sale of F1 and F2 progeny bred in India.

Although some isolated studies have identified a limited number of exotic and invasive monogenean parasites brought into India via the ornamental trade [22, 23, 52, 98–101, 103, 104], no systematic efforts targeting Monogenea have been undertaken in the country, and very few studies have been carried out elsewhere. Therefore, this study aimed to develop a standard database on the numbers, diversity, source regions, and infection parameters (prevalence and intensity) of exotic monogenean species in India on a broad scale. This was to be accomplished using a combination of both morphological and molecular taxonomy, which would allow for the achievement of the objective in a pragmatic timeframe, while overcoming their individual drawbacks. Since the reproductive capacity of monogeneans, such as *Gyrodactylus*, is positively correlated to temperature [36, 66], we also evaluated the potential risk associated with their future introductions in this new era of changing climate conditions worldwide.

## Materials and methods

### Ethics statement

Live fishes were collected, maintained, handled, and examined in accordance with the protocol approved by the animal ethical committee of the University of Lucknow, Uttar Pradesh, India (LU/AEC/ZOO/2019 and 19/I/2024/IAEC/LU).

### Study area

**Figure 1:**
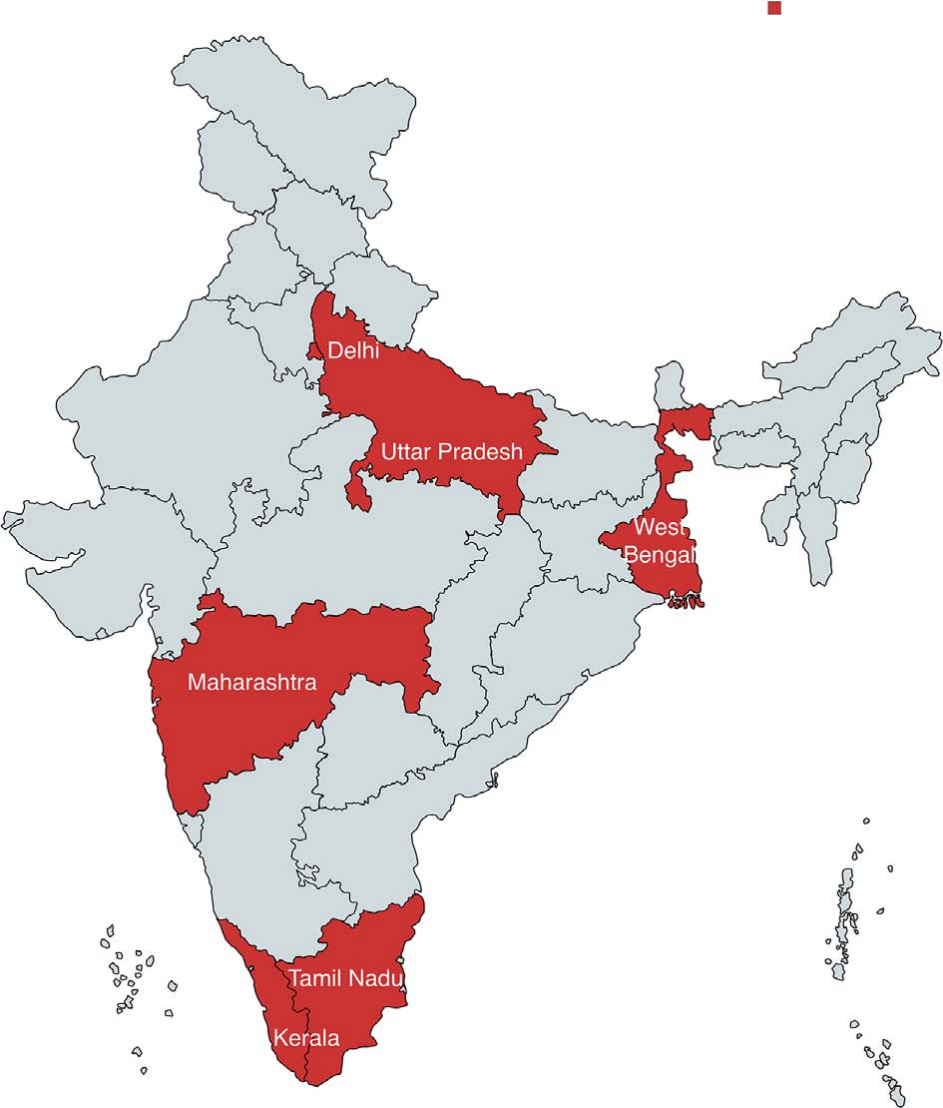
Study area map showing the collection sites (in red colour) across India (not to be scaled).

The sampling design employed to collect fishes included two approaches: fixed-station sampling and random sampling. For the fixed-station sampling, the domestic aquarium markets of the following five stations were selected: Delhi, Mumbai (Maharashtra), Kolkata (West Bengal), Kochi (Kerala), and Chennai (Tamil Nadu) (Fig. 1). These stations were selected for two reasons. First, they already have (or will be chosen to have) Government of India-designated seaports or airports for the import of exotic ornamental fish. Second, the fixed-station sampling design helped detect the true trends of abundance of both hosts and their parasites compared with the random sampling design. During the random sampling process, an attempt was made to collect the fishes from whichever stations possible across India, especially Uttar Pradesh, where it was possible to do so.

### Fish collection and examination

Between August 2019 and July 2022, 619 individuals of 27 species of ornamental fish were acquired from aquarium retailers, wholesalers, and importers. Wherever possible, live specimens were shipped to the laboratory at the University of Lucknow, UP, India on the same day they were collected after being packaged in polybags filled with water and pure oxygen. Alternatively, their gills were surgically removed and immediately fixed in hot (60 °C) 4% formalin (for morphological analysis) and 96% ethanol (for DNA analysis) before being transported. All individual fish specimens were morphologically identified using original references listed in FishBase [33]. When additional confirmation was required, the ICAR-National Bureau of Fish Genetic Resources, a leading Indian institute specialising in fish taxonomy, was consulted. Occasionally, CO1 barcoding was also utilised for identification purposes. Live fish were euthanised with an overdose of tricaine methanesulfonate (MS-222 @ 150 mg/L; Sigma Aldrich Co., St. Louis, MO, USA) until the cessation of opercular movements. Monogeneans were isolated from the gill lamellae under a stereomicroscope (Leica Microsystems, Wetzlar, Germany) using dissecting needles.

### Parasite sampling

#### Microscopy

Formalin-fixed worms were mounted in pure glycerine, Hoyer’s medium, or 10% sodium dodecyl sulphate (SDS) [110]; a few others were stained with Gomori’s trichrome, dehydrated with a graded series of ethanol, cleared in xylene, and mounted with dibutylphthalate polystyrene xylene. Additionally, ethanol-preserved worms were treated for 20–30 min at 55 °C with 1.0 μL of digestion buffer (0.1 μL of solid tissue buffer and 0.9 μL of Proteinase-K, Zymo Quick-DNA Miniprep) to digest the tissues surrounding their haptoral and reproductive sclerotised parts.

Photographs and measurements (in micrometres) of sclerotised parts, which determine the taxonomy of monogeneans [76], were obtained using a digital camera (Leica DFC7000 T) and imaging analysis software (LAS X; Leica Microsystems) attached to a light microscope (Leica DM4B). Specimens were identified morphologically using scientific literature relevant to the group (*e.g.* specialist publications and monographs), and original descriptions were consulted wherever required and possible. The photographs of haptoral sclerites of all monogenean species collected are presented here to serve as photographic vouchers, allowing parasitologists worldwide to quickly compare and identify their specimens. The prevalence (percentage of infected hosts in a sample) and mean intensity (average number of parasites per infected host in a sample) of infection were determined following the methodology outlined by Bush *et al*. [19].

#### DNA extraction, amplification, and sequencing

Ethanol-preserved monogeneans were first identified morphologically before DNA extraction. Subsequently, they were subjected to centrifugation at 12,000 ×*g* for 1 min, followed by decanting of the preservative supernatant. The genomic DNA of individual monogeneans was extracted with either the Extracta DNA Prep for PCR-Tissue (Quantabio, Beverly, MA, USA) or Zymo Quick-DNA MiniPrep kit (Zymo Research, Irvine, CA, USA). A NanoDrop 2000 (Thermo Fisher Scientific, Waltham, MA, USA) was used to determine the concentration and purity of the DNA sample before polymerase chain reaction (PCR). Portions of the ribosomal gene clusters (28S and ITS2) were amplified in an automated thermal cycler (HiMedia Laboratories, Thane, MH, India) using the previously validated primers. These nuclear ribosomal DNA (rDNA) markers were chosen because they are the most commonly sequenced and versatile genes in molecular taxonomy and phylogenetics of monogenean parasites [59, 72, 74]. Additionally, the reference sequences for most of the vouchered specimens of monogeneans were available on the National Centre for Biotechnology Information (NCBI; USA) GenBank (http://www.ncbi.nlm.nih.gov/genbank/), allowing us to confidently confirm species identities via sequencing.

Primers are detailed in Table 1, while PCR reagents and their concentrations are presented in Table 2. The amplification profile for the 28S ribosomal RNA gene followed Šimková *et al*. [89] and the amplification profile for the ITS2 region was adapted from Hahn *et al*. [39] as follows: initial denaturation at 95 °C for 3 min, then 35 cycles of denaturation at 95 °C for 30 s, annealing at 50 °C for 30 s, and extension at 72 °C for 1 min, with a final extension at 72 °C for 7 min. PCR products (2 μL) were checked for quality and length conformity on a 1.2% agarose gel pre-stained with 0.1 μL/mL of 10,000X SYBR Safe. Visualisation and documentation were done with a Bio Vision Imaging system (Vilber Lourmat, Marne-la-Vallée, France), and the most intense products were selected for sequencing. A standard 100-bp DNA ladder (HiMedia) was used to estimate the molecular weight of the amplified products.

**Table 1:** List of primers used for amplification and sequencing in this study.

Marker and Primer name	Primer sequence (5′-3′)	Reference
28S rRNA		
c1	F ACCCGCTGAATTTAAGCAT	[42]
d2	R TGGTCCGTGTTTCAAGAC	
F1	GCGAGTGAACGGAGATTAGC	[2]
R1	CCATTATTGACCGTGATGTATG	
ITS2		
ITS2	TCCTCCGCTTAGTGATA	[63]
ITS 4.5	CATCGGTCTCTCGAACG	

**Table 2:** PCR reagents in the order and concentration they were added.

Reagents	Concentration of stock solution	Volume	Final concentration
Distilled water	–	4 μL	–
Master mix	2X	10 μL	1X
Forward primer	10 μM = 10 pmols/μL	1.0 μL	0.5 pmols
Reverse primer	10 μM = 10 pmols/μL	1.0 μL	0.5 pmols
Sample DNA	–	4 μL	20 ng/μL
Total (reaction) volume	–	20 μL	–

PCR products were purified using a 1.5% agarose gel and a QIAquick PCR Purification Kit (QIAGEN, Hilden, Germany), then sequenced using Sanger sequencing on an ABI 3730xL automated sequencer (Applied Biosystems, Waltham, MA, USA). Sequencing was performed with the same primer combinations used for PCR amplification at the following commercial sequencing facilities in India: Barcode Biosciences, BioKart India, and Eurofins Genomics India.

Sequence quality control was performed with SnapGene version 5.3 (https://www.snapgene.com), using default parameters and manual curation of base-calling where necessary. Trimmed sequences were then assembled to produce contigs using the BioEdit Program [40], DNA Sequence Assembler v4 [28], and CAP3 assembly program [44]. Sequences were submitted to GenBank under the accession numbers indicated in Table 3.

**Table 3:** Summary results of BLASTn search for all monogenean species identified in the current study by gene regions (28S rDNA and ITS2) (as on December 30, 2024).

Species (GenBank Accession No.)	Hits				
	By parasite	Query cover	% identity	Accession no.	Reference
**A. 28S rRNA gene**					
***Cichlidogyrus***					
*C. tilapiae* (MZ265190)	*C. tilapiae*	100%	99.85%	MH767408	[90]
***Dactylogyrus***					
*D*. *anchoratus* (PP092478)	*D*. *anchoratus*	93%	99.85%	MF975788	[82]
	*D*. *anchoratus*	100%	99.72%	MT997190	Unpublished
	*D*. *anchoratus*	100%	99.02%	KY863555	[9]
	*D. formosus*	100%	100%	PP825049	Unpublished
* D*. *baueri* (PQ216314)	*D*. *baueri*	100%	100%	PP188041	Unpublished^1^
* D*. *extensus* (PP092479)	*D*. *extensus*	100%	99.87%	LC764381	[71]
	*D*. *extensus*	100%	99.74%	AY553629	[112]
* D*. *formosus* (PP092481)	*D*. *formosus*	97%	100%	MG792984	[10]
* D. intermedius* (PQ216315)	*D. intermedius*	97%	100%	OQ944102	[79]
* D*. *lampam* (PQ216363–PQ216370)	*D*. *lampam*	99%	99.15%	OR077123	[67]
* D*. *vastator* (PP092480)	*D*. *vastator*	100%	100%	KY201106	[9]
* D. minutus* (OK037582)	*Dactylogyrus* sp.	100%	100%	MK335464	Unpublished
	*D. minutus*	95%	98.46%	MF926269	[82]
* D. volfi* (PQ838311–PQ838314)	*D. zandti*	97%	85.53%	OP595735	Unpublished
***Diaphorocleidus***					
* D*. *armillatus* (PQ269273)	*D. neotropicalis*	91%	88.30%	MZ408906	[116]
***Gussevia***					
* G. asota* (ON614225)	*G. asota*	100%	100%	MG596661	[114]
***Heteronchocleidus***					
* Heteronchocleidus* sp. (PQ278851)	*H. buschkieli*	80%	97.77%	AY841876	[27]^2^
***Heteropriapulus***					
* H*. *heterotylus* (PQ269164)	*H*. *heterotylus*	98%	99.53%	MF116370	[1]
***Metahaliotrema***					
* M*. *mizellei* (PQ404355–PQ404357)	*M*. *mizellei*	93%	99.36%	DQ157647	[113]
***Mymarothecium***					
* Mymarothecium* sp. (PQ283971–PQ283972)	*M. viatorum*	95%	85.80%	PQ220219	Present study
	*M. viatorum*	80%	84.96%	MH843723	[68]
* M. viatorum* (PQ220219–PQ220220, PQ220141)	*M. viatorum*	94%	100%	MH843723	[68]
***Sciadicleithrum***					
* S*. *iphthimum* (PQ308992)	*S*. *iphthimum*	67%	100%	OQ822829	[52]
	*S*. *iphthimum*	80%	97.01%	OQ800931	[52]
***Trianchoratus***					
* T*. *trichogasterium* (PQ249168)	*T*. *acleithrium*	100%	98.46%	PQ249166	Present study
* T*. *acleithrium* (PQ249166–PQ249167)	*T*. *trichogasterium*	91%	98.46%	PQ249168	Present study
***Urocleidoides***					
*Urocleidoides* sp. (PQ824969– PQ824970)	*U. vanini*	71%	85.01%	OR270736	[84]
**B. ITS2 region**					
***Gyrodactylus***					
*G*. *bullatarudis* (OR405269)	*G*. *bullatarudis*	100%	99.78%	AY692024	[20]
*G*. *gurleyi* (MZ356513)	*G*. *gurleyi*	100%	99.62%	KC922453	[56]
*G*. *kobayashii* (MZ356536)	*G*. *kobayashii*	100%	100%	MF356251	[105]
*G*. *sprostonae* (MZ356537)	*G*. *sprostonae*	92%	100%	AY278044	[117]
*Gyrodactylus* sp.1 (MZ358889)	*G. banmae*	93%	95.89%	MW353802	[45]

1 This is the only record of 28S rDNA of *D*. *baueri* on GenBank.

2 This is the only record of 28S rDNA of *Heteronchocleidus* on GenBank.

#### Molecular species identification

The contigs were subjected to a BLAST search against the nt core database of NCBI to confirm the initial morphological identification of species. Species were identified based on a comparison of the query sequence to the subject sequences from the database with the highest index of identity, highest score, and lowest e-value.

## Results and discussion

### Species diversity (Table 4, Figs. 2–4)

#### Morphological detection

A total of 619 individual fishes belonging to 27 species, 24 genera, nine families, and seven orders were collected and examined for monogenean parasites. Of the 27 sampled host species, 17 (63%) were found to carry Monogenea. Morphological diagnoses identified 26 monogenean species. Among them, 22 were identified to the species level. Four additional records could not be identified at the species level. These may represent species new to science and will be investigated in greater depth in future work. Specifically, these species are *Heteronchocleidus* sp. from *Betta splendens*, *Mymarothecium* sp. from *Metynnis hypsauchen*, *Gyrodactylus* sp. from *Cyprinus carpio*, and *Urocleidoides* sp. from *Xiphophorus helleri*. Ten (37%) fish species were found to be free of monogenean infection. All identified species belonged to the following 12 genera: *Cichlidogyrus*, *Dactylogyrus*, *Diaphorocleidus*, *Gussevia*, *Gyrodactylus*, *Heteronchocleidus*, *Heteropriapulus*, *Metahaliotrema*, *Mymarothecium*, *Sciadicleithrum*, *Trianchoratus*, and *Urocleidoides*. *Dactylogyrus* had the most species with nine, followed by *Gyrodactylus* with five. The most parasitised fish family was Cyprinidae (13 species), followed by Cichlidae (three species) and Helostomatidae, Poeciliidae, and Serrasalmidae (two species each).

**Figure 2:**
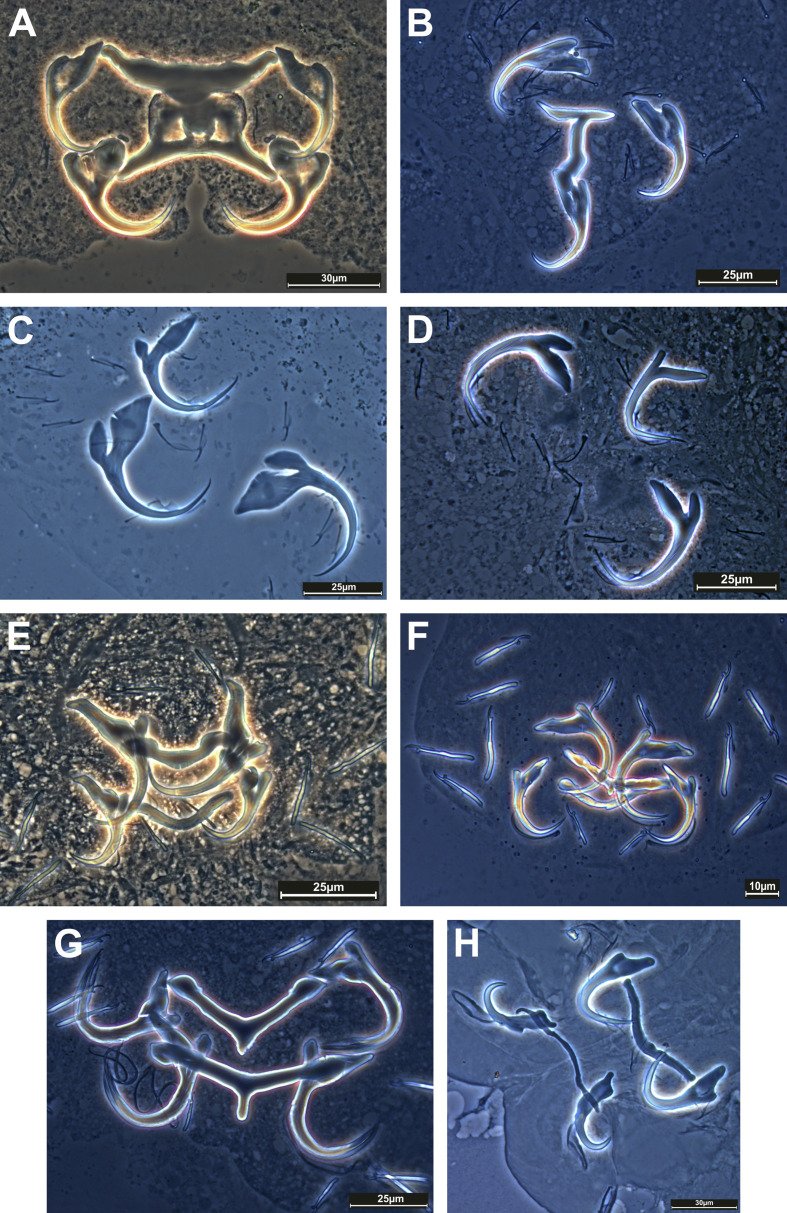
Phase-contrast images of haptoral armaments (anchor-bar complex and hooks) of monogenean species: A) *Metahaliotrema mizellei* Venkatanarasaiah, 1981; B) *Heteronchocleidus* sp.; C) *Trianchoratus acleithrium* Price & Berry, 1966; D) *Trianchoratus trichogasterium* Lim, 1986; E) *Diaphorocleidus armillatus* Jogunoori, Kritsky & Venkatanarasaiah, 2004; F) *Mymarothecium* sp.; G) *Mymarothecium viatorum* Boeger, Piasecki & Sobecka, 2002; H) *Gussevia asota* Kritsky, Thatcher & Boeger, 1989 [Reproduced from Tripathi and Matey [104] with permission from the copyright holder].

**Figure 3:**
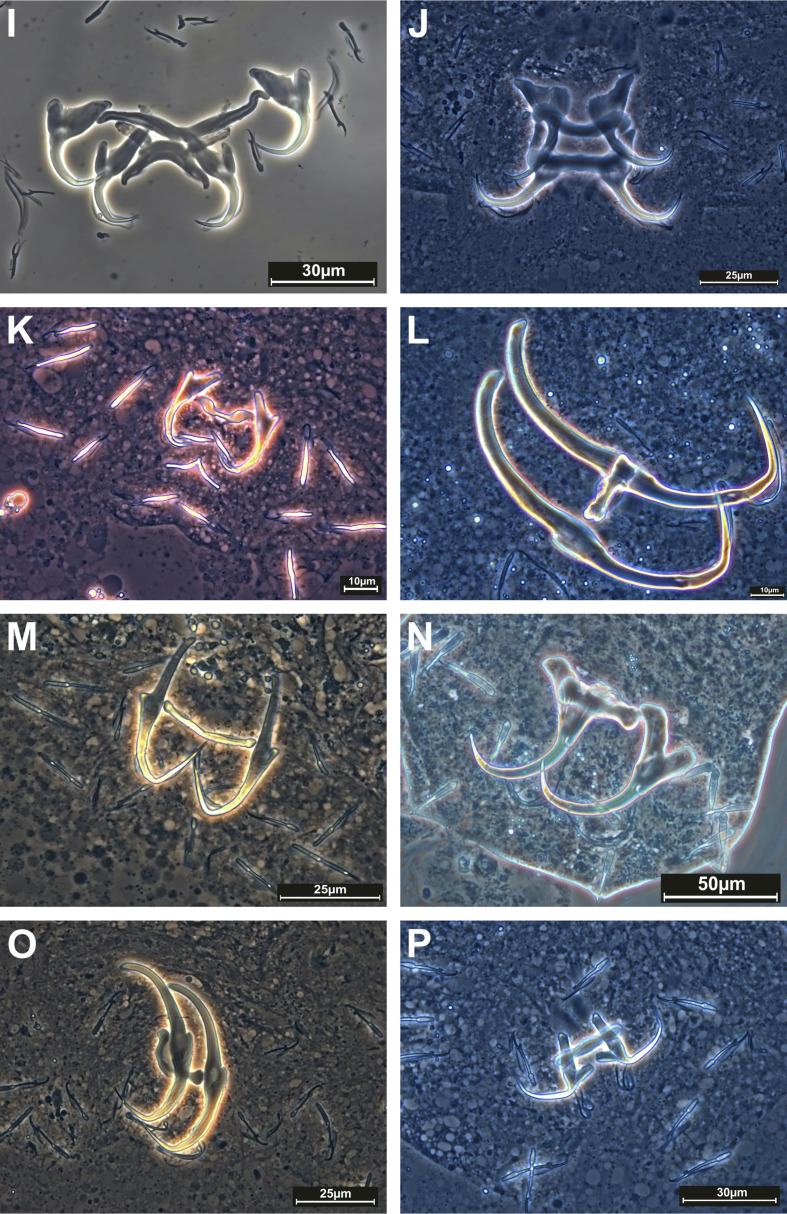
Phase-contrast images of haptoral armaments (anchor-bar complex and hooks) of monogenean species: I) *Cichlidogyrus tilapiae* Paperna, 1960; J) *Sciadicleithrum iphthimum* Kritsky, Thatcher & Boeger, 1989; K) *Dactylogyrus lampam* Lim, 1992; L) *Dactylogyrus anchoratus* (Dujardin, 1845) Wagener, 1857; M) *Dactylogyrus baueri* Gussev, 1955; N) *Dactylogyrus extensus* Mueller & Van Cleave, 1932; O) *Dactylogyrus formosus* Kulwiec, 1927; P) *Dactylogyrus intermedius* Wegener, 1910.

**Figure 4:**
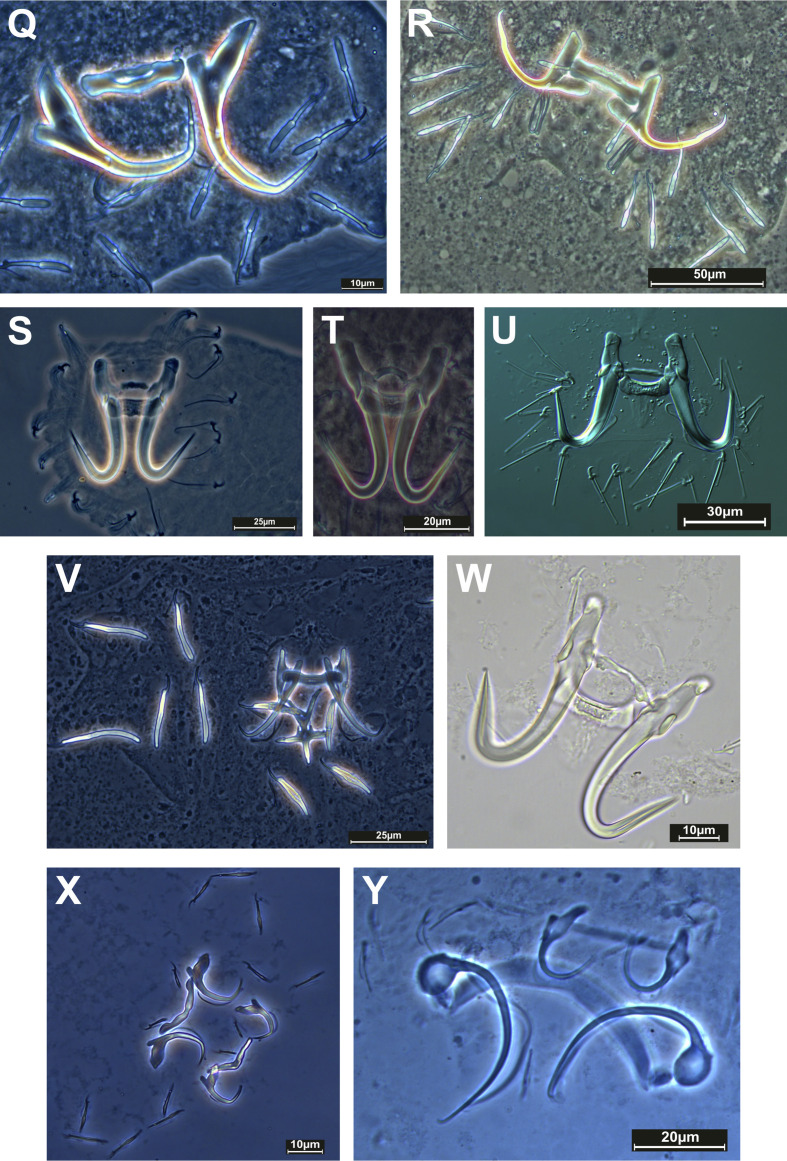
Phase-contrast images of haptoral armaments (anchor-bar complex and hooks) of monogenean species: Q) *Dactylogyrus minutus* Kulwiec, 1927; R) *Dactylogyrus vastator* Nybelin, 1924; S) *Gyrodactylus gurleyi* Price 1937; T) *Gyrodactylus kobayashii* Hukuda, 1940; U) *Gyrodactylus* sp.; V) *Dactylogyrus volfi* Lucky, 1970; W) *Gyrodactylus bullatarudis* Turnbull, 1956; X) *Urocleidoides* sp.; Y) *Heteropriapulus heterotylus* (Jogunoori, Kritsky & Venkatanarasaiah, 2004) Kritsky, 2007.

**Table 4:** Host-parasite list indicating the exotic ornamental fish examined and monogenean parasite found (+ve indicates infection found; −ve indicates infection not found). *New host record for monogenean parasites; #First record in India.

Fish host and classification	Sample size	Native locality	Infection status	Monogenean species
Order: Acanthuriformes				
Family: Scatophagidae				
1. *Scatophagus argus* (Linnaeus, 1766) (Spotted scat)	15	Indo–Pacific	+ve	*Metahaliotrema mizellei* Venkatanarasaiah, 1981
Order: Anabantiformes				
Family: Helostomatidae				
2. *Betta splendens* Regan, 1910* (Siamese fighting fish)	10	Asia	+ve	*Heteronchocleidus* sp.
3. *Helostoma temminckii* Cuvier, 1829 (Kissing gourami)	20	Asia	+ve	*Trianchoratus acleithrium* Price & Berry, 1966
Family: Osphronemidae				
4. *Osphronemus goramy* Lacepède, 1801 (Giant gourami)	22	Asia	−ve	
5. *Trichopodus trichopterus* (Pallas, 1770) (Three spot gourami)	26	Asia	+ve	*Trianchoratus trichogasterium* Lim, 1986
Order: Characiformes				
Family: Characidae				
6. *Gymnocorymbus ternetzi* (Boulenger, 1895) (Black tetra)	25	S. America	+ve	*Diaphorocleidus armillatus* Jogunoori, Kritsky & Venkatanarasaiah, 2004
7. *Hemigrammus erythrozonus* Durbin, 1909 (Glowlight tetra)	15	S. America	−ve	
Family: Serrasalmidae				
8. *Metynnis hypsauchen* (Muller & Troschel, 1844)* (Silver dollar)	20	S. America	+ve	*Mymarothecium* sp.
9. *Piaractus brachypomus* (Cuvier, 1818) (Pirapitinga)	24	S. America	+ve	*Mymarothecium viatorum* Boeger, Piasecki & Sobecka, 2002#
Order: Cichliformes				
Family: Cichlidae				
10. *Astronotus ocellatus* (Agassiz, 1831) (Oscar)	12	S. America	+ve	*Gussevia asota* Kritsky, Thatcher & Boeger 1989#
11. *Chindongo socolofi* (Johnson, 1974)* (Pindani)	22	Africa	+ve	*Cichlidogyrus tilapiae* Paperna, 1960#
12. *Hemichromis fasciatus* Peters, 1857 (Banded jewelfish)	15	Africa	−ve	
13. *Mikrogeophagus ramirezi* (Myers & Harry, 1948) (Ram cichlid)	23	S. America		−ve
14. *Pterophyllum scalare* (Schultze, 1823) (Freshwater angelfish)	10	S. America	+ve	*Sciadicleithrum iphthimum* Kritsky, Thatcher & Boeger, 1989
15. *Thorichthys meeki* Brind, 1918 (Firemouth cichlid)	18	C. America	−ve	
Order: Cypriniformes				
Family: Cyprinidae				
16. *Barbonymus altus* (Günther, 1868) (Red tailed tinfoil)	28	Asia	+ve	*Dactylogyrus lampam* Lim, 1992#
17. *Carassius auratus* (Linnaeus, 1758) (Goldfish)	63	Asia	+ve	*Dactylogyrus anchoratus* (Dujardin, 1845) Wagener, 1857
				*Dactylogyrus baueri* Gussev, 1955#
				*Dactylogyrus extensus* Mueller & Van Cleave, 1932#
				*Dactylogyrus formosus* Kulwiec, 1927#
				*Dactylogyrus intermedius* Wegener, 1910#
				*Dactylogyrus minutus* Kulwiec, 1927
				*Dactylogyrus vastator* Nybelin, 1924
				*Gyrodactylus gurleyi* Price 1937#
				*Gyrodactylus kobayashii* Hukuda, 1940#
18. *Cyprinus carpio* Linnaeus, 1758 (Common carp)	35	Asia	+ve	*Dactylogyrus anchoratus* (Dujardin, 1845), Wagener, 1857
				*Dactylogyrus extensus* Mueller & Van Cleave, 1932
				*Dactylogyrus minutus* Kulwiec, 1927#
				*Dactylogyrus vastator* Nybelin, 1924
				*Gyrodactylus sprostonae* Ling, 1962#
				*Gyrodactylus* sp.
19. *Epalzeorhynchos frenatum* (Fowler, 1934) (Rainbow sharkminnow)	22	Asia	−ve	
20. *Pethia conchonius* (Hamilton, 1822) (Rosy barb)	15	Asia	−ve	
21. *Puntigrus tetrazona* (Bleeker, 1855) (Sumatra barb)	15	Asia	+ve	*Dactylogyrus volfi* Lucky, 1970#
Order: Cyprinodontiformes				
Family: Poeciliidae				
22. *Poecilia latipinna* (Lesueur, 1821) (Sailfin molly)	25	N. America	−ve	
23. *Poecilia reticulata* Peters, 1859 (Guppy)	30	N. America	+ve	*Gyrodactylus bullatarudis* Turnbull, 1956
24. *Poecilia sphenops* Valenciennes, 1846 (Molly)	25	C. & S. America	−ve	
25. *Xiphophorus hellerii* Heckel, 1848 (Green sword tail)	27	N. & C. America	+ve	*Urocleidoides* sp.
26. *Xiphophorus maculatus* (Gunther, 1866) (Southern platyfish)	40	N. & C. America	−ve	
Order: Siluriformes				
Family: Loricariidae				
27. *Pterygoplichthys disjunctivus* (Weber, 1991) (Vermiculated sailfin catfish)	17	S. America	+ve	*Heteropriapulus heterotylus* (Jogunoori, Kritsky & Venkatanarasaiah, 2004) Kritsky, 2007

#### Molecular detection (Table 3)

The PCR amplification and sequencing of the 28S rDNA or ITS2 region was successful for all 26 monogenean species recovered. Thirty-seven sequences were generated and submitted to GenBank. In most cases, molecular data supported our morphological diagnoses, with best matches generally exhibiting over 99% sequence similarity to previously deposited sequence records of the species in question, confirming the efficacy of molecular species identification.

However, two examples of the potential pitfalls of purely molecular diagnoses, especially in difficult taxonomic groups such as Monogenea, need further discussion. First, while the sequence we obtained for *D. anchoratus* typically confirmed our morphological species identification (multiple records of *D. anchoratus* with >99% similarity to ours), the BLAST search also returned a match to a record of *D. formosus* (PP825049.1, 100% similarity). The latter record may be a misidentification since the morphological diagnosis supporting it cannot be verified in contrast to ours (as it was deposited as a direct GenBank submission independent of publication) (see Fig. 3L), and it shows about 3% sequence divergence to other representatives of *D. formosus*.

Second, our sequence for *T. trichogasterium* (PQ249168) showed 98.78% and 97.28% similarity to records of *T. acleithrium* (HQ719214.1) and *T. trichogasterium* (HQ719217.1), respectively. As with the previous case, these latter records resulted from direct GenBank submissions and cannot be verified morphologically. Thus, we regard our record of *T. trichogasterium* as correct, and we consider the only other 28S record for the species representing a misidentification.

The molecular diagnosis of *Dactylogyrus volfi* from *Puntigrus tetrazona* (Bleeker, 1855) (Cyprinidae), and *Urocleidoides* sp. from *Xiphophorus hellerii* Heckel, 1848 (Poeciliidae) was challenging. *Dactylogyrus volfi* was first described by Lucky (1970) on *Barbus tetrazona* (now *P*. *tetrazona*) in Czechoslovakia. Later, Borisov (2013) recorded it from the same fish host in Bulgaria. *Puntigrus tetrazona* is native to Sumatra and Borneo in Indonesia (Asia), and is currently known to host only *D. volfi* as a monogenean parasite. Our specimens of *D. volfi* showed morphological consistency with Lucky’s (1970) and Borisov’s (2013) descriptions.

Although the sequencing results of all four isolates of *D. volfi* matched exclusively with *Dactylogyrus* spp. in the NCBI database, one isolate indicated a significant sequence divergence of 13.50–17.74% from the other three, which were identical. Although DNA contamination cannot be excluded as the cause of this result, the high-quality sequencing chromatogram for the deviant isolate strongly suggests a previously unrecognised cryptic species within the population of *D. volfi* in India, with little or no morphometric difference to distinguish them. Of note, no corresponding sequence of *D. volfi* is available in the NCBI nucleotide database to independently confirm the specific status of our specimens from *P*. *tetrazona* as *D. volfi*. Thus, genetic monitoring is crucial in detecting an overlooked cryptic monogenean species. Further verification with additional nuclear and mitochondrial gene markers is recommended.

*Xiphophorus helleri*, a native to tropical Mexico, hosts a single dactylogyrid species, *Urocleidoides vaginoclaustrum*, which Jogunoori, Kritsky, and Venkatanarasaiah (2004) described from India. BLAST analysis for the parasite recovered from *X*. *hellerii* identified a “top hit” (smallest E-value) with 85.01% similarity to *Urocleidoides vanini* Santos Neto & Domingues, 2023 from *Erythrinus erythrinus* (Bloch & Schneider, 1801) in Brazil. However, it also had a “best hit” (highest % identity) with 93.21% similarity to *Onchocleidus principalis* Mizelle, 1936 from *Micropterus salmoides* (Lacépède, 1802) in Portugal. Nevertheless, the specimens displayed all key morphological characteristics of *Urocleidoides* Mizelle & Price, 1964, including hook-shaped vaginal sclerite and coiled MCO with counter-clockwise rings. Of note, the NCBI database contains no reference sequence for *U*. *vaginoclaustrum*. Thus, based on similar comparative morphological characteristics, the identical original host, and the high host specificity of monogenean parasites, combined with BLAST results, we provisionally classify our specimens as *Urocleidoides* sp. until additional material is collected.

For the species *D. armillatus*, no sequence of the 28S gene was hitherto represented on GenBank. At present, the closest match to our record was *D. neotropicalis* Zago, Franceschini, Abdallah, Müller, Azevedo & da Silva, 2021 (MZ408906.1), with 88.30% sequence similarity. The genus *Heteronchocleidus* is currently represented on GenBank by only one species, *H. buschkieli* Bychowsky, 1957, and the record in question (AY841876.1) has 97.77% similarity to our putative *Heteronchocleidus* sp., validating our generic diagnosis but yielding no further insights.

#### Infection parameters (Table 5)

The presence of a parasite and its infection parameters in a host are the key factors in determining the parasite’s ability to enter and establish itself in the receiving country [48]. Of the 619 individuals from 27 species of exotic ornamental fish examined, 240 individuals (39%) from 17 species (63%) were confirmed to be infected with 26 species of monogenean parasites. The most parasitised fish family was Cyprinidae (13 species), followed by Cichlidae (three species) and Helostomatidae, Poeciliidae, and Serrasalmidae (two species each). Only two host species (12%) harboured multiple species infections (*C*. *auratus* and *C. carpio*)*,* while 15 fishes (88%) carried one monogenean species each. The observed prevalence of monogeneans collected from certain representatives of Characiformes (*G. ternetzi, P. brachypomus*), Cichliformes (*A. ocellatus*), and Cypriniformes (*B. altus, C. auratus*, and *C. carpio*) was 100% across all the sub-regions of India where fish were collected.

**Table 5:** Infection parameters of monogenean parasites from exotic Indian freshwater fish collected in 2019–2022. At least ten fish were examined for each species.

Fish hosts	NE	Parasite species	NI	NM	P (%)	MI
1. *Scatophagus argus*	15	*Metahaliotrema mizellei*	3	12	20	4
2. *Betta splendens*	10	*Heteronchocleidus* sp.	4	8	40	2
3. *Helostoma temminckii*	20	*Trianchoratus* acleithrium	3	8	15	2.6
4. *Osphronemus goramy*	22	–	0	0	0	0
5. *Trichopodus trichopterus*	26	*Trianchoratus trichogasterium*	10	85	38.46	8.5
6. *Gymnocorymbus ternetzi*	25	*Diaphorocleidus armillatus*	25	860	100	34.4
7. *Hemigrammus erythrozonus*	15	–	0	0	0	0
8. *Metynnis hypsauchen*	20	*Mymarothecium* sp.	5	38	25	7.6
9. *Piaractus brachypomus*	24	*Mymarothecium viatorum*	24	720	100	30
10. *Astronotus ocellatus*	12	*Gussevia asota*	12	600	100	50
11. *Chindongo socolofi*	22	*Cichlidogyrus tilapiae*	5	31	22.72	6.2
12. *Hemichromis fasciatus*	15	–	0	0	0	0
13. *Mikrogeophagus ramirezi*	23	–	0	0	0	0
14. *Pterophyllum scalare*	10	*Sciadicleithrum iphthimum*	4	12	33.33	3
15. *Thorichthys meeki*	18	–	0	0	0	0
16. *Barbonymus altus*	28	*Dactylogyrus lampam*	28	468	100	16.7
17. *Carassius auratus*	63^1^	*Dactylogyrus anchoratus*	46	292	73	6.3
		*Dactylogyrus baueri*	20	80	31.7	4
		*Dactylogyrus extensus*	20	180	31.7	9
		*Dactylogyrus formosus*	40	220	63.4	5.5
		*Dactylogyrus intermedius*	30	300	47.6	10
		*Dactylogyrus minutus*	15	50	23.8	3.3
		*Dactylogyrus vastator*	58	636	92	10.9
		*Gyrodactylus gurleyi*	12	64	33.3	5.3
		*Gyrodactylus kobayashii*	9	56	14.28	6.2
18. *Cyprinus carpio*	35	*Dactylogyrus anchoratus*	20	140	57.14	7
		*Dactylogyrus extensus*	8	280	22.85	35
		*Dactylogyrus minutus*	15	180	100	12
		*Dactylogyrus vastator*	20	240	57.14	12
		*Gyrodactylus sprostonae*	04	25	22.85	6.25
		*Gyrodactylus* sp.1	06	15	34.28	2.5
19. *Epalzeorhynchos frenatum*	22	–	0	0	0	0
20. *Pethia conchonius*	15	–	0	0	0	0
21. *Puntigrus tetrazona*	15	*Dactylogyrus volfi*	4	8	26.66	4
22. *Poecilia latipinna*	25	–	0	0	0	0
23. *Poecilia reticulata*	30	*Gyrodactylus bullatarudis*	2	6	6.6	3
24. *Poecilia sphenops*	25	–	0	0	0	0
25. *Xiphophorus hellerii*	27	*Urocleidoides* sp.	3	10	11.11	3.3
26. *Xiphophorus maculatus*	40	–	0	0	0	0
27. *Pterygoplichthys disjunctivus*	17	*Heteropriapulus heterotylus*	10	48	58.82	4.8
Total	619		240	5672		

NE is the number of fish examined; NI is the number of fish infected; NM is the number of monogenean parasites recovered; P is the prevalence in percentage; MI is the mean intensity.

1 All *C. auratus* sampled were found to be infected with one or another species of monogenean parasites.

The cyprinid *C*. *auratus* was the fish with the highest parasite species richness, with nine taxa, followed by another cyprinid (*C*. *carpio*), with six taxa. This large symbiota (host-parasite complex) [34] of *C*. *auratus*, including seven *Dactylogyrus* spp. and two *Gyrodactylus* spp., is concerning because the concept of the pathobiome [8] suggests that diseases are caused by symbionts rather than a single principal agent. Four species of *Dactylogyrus* (*D. anchoratus, D*. *extensus, D*. *minutus*, and *D*. *vastator*) were found in two fish species (*C*. *auratus* and *C*. *carpio*), indicating that more closely related fish host species were more likely to be associated with the same parasite species. In fact, the monophyly of *Dactylogyrus* spp. of these two cyprinid hosts has already been suggested by Šimková *et al*. [88, 89] and, more recently, Tripathi *et al*. [103].

The high prevalence of co-translocated monogeneans in post-quarantine populations and captive-reared exotic ornamental fish species is alarming, given that they were most likely to be exposed to antiparasitic treatments in quarantine facilities at airports as well as culture ponds and in pet shops. This suggests either that monogeneans are resistant to more commonly used anti-parasitic medications or that the owners of these accomplishments are unaware of monogeneans, which prevents these worms from receiving appropriate treatment.

#### New host and geographic records (Table 4)

Three fish were found in the first host records for monogenean parasites ever reported: *C*. *socolofi* for *C*. *tilapiae* Paperna, 1960, *M*. *hypsauchen* for *Mymarothecium* sp., and *B*. *splendens* for *Heteronchocleidus* sp.

India provides new geographic records for the following 13 known monogeneans identified at the species level: *C*. *tilapiae*, *D*. *baueri*, *D*. *extensus*, *D*. *formosus*, *D*. *intermedius*, *D*. *lampam*, *D*. *minutus*, *D*. *volfi*, *G*. *gurleyi*, *G*. *kobayashii*, *G*. *sprostonae*, *G*. *asota*, and *M*. *viatorum*. In addition, the following three monogeneans were identified at the generic level and may represent new species: unknown spp. of *Gyrodactylus*, *Heteronchocleidus*, and *Mymarothecium*. Given the vast diversity of monogeneans (>7,000 known species) [37], the discovery of a new species is not surprising, especially when new hosts are examined or known hosts are examined from a new geographic locality.

#### Parasite lost (Table 6)

Introduced species often carry half of the number of parasite species reported in native populations; this could be due to a variety of factors, including a lower risk of parasite introduction with exotic species [97]. Our research supports this viewpoint. For instance, a comprehensive review of the literature identified four monogenean species that were described from *A. ocellatus*, three described from *B*. *altus*, 26 described from *C. auratus*, 38 described from *C. carpio*, four described from *P*. *disjunctivus*, *P*. *reticulata*, and *S*. *argus* each, and eight described from *X*. *helleri*. Nonetheless, we found nine monogenean species in *C. auratus*, six in *C. carpio*, and just one each in *A. ocellatus*, *B*. *altus*, *P*. *disjunctivus*, *P*. *reticulata*, *S*. *argus*, and *X*. *helleri*.

**Table 6:** Known host-parasite association (compiled from contributions in [54, 24, 41, 46, 76, 35, 55, 70, 105]) NOT found in this study.

Host	Monogenean parasites
*Astronotus ocellatus* (Agassiz, 1831)	*Gussevia astronoti* Kritsky, Thatcher & Boeger, 1989; *G*. *rogersi* Kritsky, Thatcher & Boeger, 1989; *Gyrodactylus cichlidarum* Paperna, 1968.
*Barbonymus altus* (Günther, 1868)	*Dactylogyrus tapienensis* Chinabut & Lim, 1993; *D. viticulus* Chinabut & Lim, 1993.
*Carassius auratus* (Linnaeus, 1758)^1^	*Dactylogyrus achmerowi* Gussev, 1955; *D*. *arcuatus* Yamaguti, 1942; *D. crassus* Kulweic, 1927; *D. dogieli* Gussev, 1953; *D. dulkeiti* Bychowsky, 1936; *D. falciformis* Achmerov, 1952; *D. inexpectatus* Izjumova in Gussev, 1955; *D. intermedioides* Gussev, Jalali & Molnar, 1993; *D. spiralis* Yamaguti, 1942; *D. wegeneri* Kulweic, 1927
	*Gyrodactylus elegans* von Nordmann, 1832; *G. katharineri* Malmberg, 1964; *G. longoacuminatus* Zitnan, 1964; *G. medius* Kathariner, 1895; *G. shulmani* Ling, 1962; *G*. *sprostonae* Ling, 1962, *G. carassii* Malmberg, 1957.
*Cyprinus carpio* Linnaeus, 1758	*Dactylogyrus achmerovi* Gussev, 1955; *D. baueri* Gussev, 1955; *D. biwaensis* Ogawa & Egusa, 1982; *D. crassus* Kulweic, 1927; *D. falciformis* Achmerov, 1952; *D. formosus* Kulweic, 1927; *D. inexpectatus* Izjumova in Gussev, 1955; *D. intermedius* Wegener, 1910; *D. lopuchinae* Jukhimenko, 1981; *D. molnari* Ergens & Dulmaa, 1969; *D. mrazeki* Ergens & Dulmaa, 1969; *D. sahuensis* Ling in Chen et al., 1973; *D. takahashii* Ogawa & Egusa, 1982; *D. tuba* Linstow, 1878; *D. yinwenyingae* Gussev in Bykhovskaya-Pavlovskaya et al., 1962.
	*Gyrodactylus bimicroforatus* Jin, 1993; *G. carassii* Malmberg, 1957; *G. cyprini* Diarova, 1964; *G. derjavini* Mikhailov, 1975; *G. fairporti* Van Cleave, 1921; *G. gurleyi* Price, 1937; *G. katharineri* Malmberg, 1964; *G. kherulensis* Ergens, 1974; *G. longoacuminatus* Zitnan, 1964; *G. medius* Kathariner, 1895; *G. nagibinae* Gusev, 1962; *G. ophiocephali* Gusev, 1955; *G. osoblahensis* Ergens, 1963; *G. procerus* Lux, 1990; *G. shulmani* Ling, 1962; *G. stankowici* Ergens, 1970; *G. vimbi* Shulman, 1954.
*Pterygoplichthys disjunctivus* (Weber, 1991)	*Trinigyrus peregrinus* Nitta & Nagasawa, 2016;
	*Unilatus brittani* Mizelle, Kritsky & Crane, 1968; *Unilatus unilatus* Mizelle & Kritsky, 1967.
*Poecilia reticulata* Peters, 1859	*Gyrodactylus poeciliae* Harris & Cable, 2000; *G. turnbulli* Harris, 1986; *Urocleidoides reticulatus* Mizelle & Price, 1964.
*Poecilia sphenops* Valenciennes, 1846	*Gyrodactylus bullatarudis* Turnbull, 1956; *G. costaricensis* Kritsky & Fritts, 1970.
*Scatophagus argus* (Linnaeus, 1766)	*Metahaliotrema scatophagi* Yamaguti, 1953; *Metahaliotrema yamagutii* Mizelle & Price, 1964; *Metahaliotrema ypsilocleithrum* Kritsky, Nguyen, Ha & Heckman, 2016.
*Xiphophorus hellerii* Heckel, 1848	*Gyrodactylus apazapanensis* García-Vásquez, Razo-Mendivil & Rubio-Godoy, 2015; *G. bullatarudis* Turnbull, 1956; *G. jarocho* Rubio-Godoy, Paladini, García-Vásquez & Shinn, 2010; *G. pseudobullatarudis* García-Vásquez, Razo-Mendivil & Rubio-Godoy, 2015; *G. rasini* Lucký, 1973; *G. xtachuna* García-Vásquez, Razo-Mendivil & Rubio-Godoy, 2015; *Urocleidoides vaginoclaustrum* Jogunoori, Kritsky & Venkatanarasaiah, 2004.

1 The majority of these parasites were originally described from *Carassius auratus gibelio*, which has now split into two species, *Carassius auratus* and *Carassius gibelio*. This makes it practically impossible to distinguish the monogeneans of these two different species based on published work alone.

Similarly, several fish (*Pethia conchonius*, *Poecilia latipinna*, and *Poecilia sphenops*) which are known to harbour monogeneans in their native habitat, were not found to be infected in our investigation. This absence of monogeneans in our study, compared to those found naturally on their hosts throughout their native range, may be an artefact due to insufficient sampling or the real phenomenon in which they were not co-transported to India with their exotic host. However, any chance of sampling bias should have been eliminated due to the large, random, and long-term sample size of our study. Therefore, it is likely that this absence is real and that the absentee species failed to be co-transported with their host (at least until now). We suppose that these fish samples were imported into India from a specific geographic region that, by chance, was infected with only selected species (missing the boat) [60], which we were able to recover in India.

#### Biogeographical patterns in host-parasite associations (Tables 4 and 7, Fig. 5)

Geographical structure patterns (from local to global scales) in multispecies host-parasite assemblages explain future spill-over and spillback by parasites. However, identifying the country of origin of imported fish (and parasites) in the ornamental fish trade is challenging due to frequent trans-shipment and relabelling [6, 14]. For example, species labelled as originating from Singapore, the largest ornamental fish exporter in the world [115], are often collected from other countries [13] before being re-exported. Therefore, we indirectly evaluated the geographic location of fish (and parasites) by considering their native distribution range based on current country-level geographic borders.

**Figure 5:**
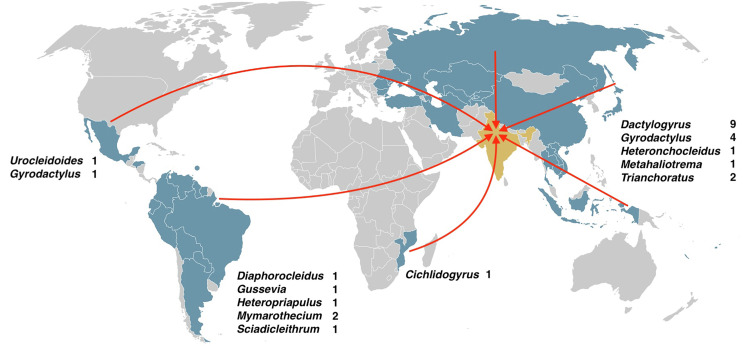
Schematic diagram representing the geographic distribution of co-introduced monogeneans recovered in India, 2019–2022.

**Table 7:** An overview of the native geographical regions of ornamental fish imported into India, together with their monogenean parasites.

Geographical region	FIM	FIN	MSR
Asia (including Indo–Pacific)	11	8	17
South America	8	6	6
North America	2	1	1
Africa	2	1	1
North & Central America	2	1	1
Central & South America	1	–	–
Central America	1	–	–
Total	26	17	26

FIM (No. of fish species imported), FIN (No. of fish species infected), and MSR (No. of monogenean species recovered).

The native distribution of collected fish from FishBase [33] indicates that the native ranges of most fish were in Asia, including the Indo–Pacific region (40.74%, *n* = 11), followed by South America (29.62%, *n* = 8), North America (7.40%, *n* = 2), Africa (7.40%, *n* = 2), North and Central America (7.40%, *n* = 2), South and Central America (3.70%, *n* = 1), and Central America (3.70%, *n* = 1). Concerning parasites’ specific heterogeneity, the majority of co-introduced parasite species originated from Asia (65.38%, *n* = 17), followed by South America (23.07%, *n* = 6), North and Central America (7.69%, *n* = 2), and Africa (3.5%, *n* = 1).

This distribution pattern reflects the numerical dominance of *Dactylogyrus* and *Gyrodactylus* species (nine and five, respectively) infecting cyprinid fishes from Asian countries. It also indicates that the total species richness of parasites in local host communities often correlates positively with the species richness of hosts. However, regarding generic heterogeneity, the majority of co-introduced parasite genera originated from Asia (41.66%, *n* = 5; *Dactylogyrus*, *Gyrodactylus*, *Heteronchocleidus*, *Metahaliotrema*, and *Trianchoratus*) and South America (41.66%, *n* = 5; *Diaphorocleidus*, *Gussevia*, *Heteropriapulus*, *Mymarothecium*, and *Sciadicleithrum*), followed by North and Central America (16.66%, *n* = 2; *Gyrodactylus* and *Urocleidoides*) and Africa (8.33%, *n* = 1; *Cichlidogyrus*).

#### Potentially invasive monogeneans (Table 8)

Of the 27 fish species studied, at least 10 are invasive in Indian waters. While parasite sampling of these fishes from wild waters remains to be done in the future, we collected two specimens of *C. carpio* and five specimens of *Oreochromis niloticus* (Linnaeus, 1758) from the river Ghaghra in northern India at the time of writing. However, we found only one monogenean species from each (*D. minutus* and *Cichlidogyrus sclerosus* Paperna and Thurston, 1969, respectively). This low species diversity of monogeneans may be due to the small sample size. It could also be explained by the “enemy release concept”, which holds that parasitism is substantially lower in introduced areas of invasive species than in conspecific populations in native areas [97]. Therefore, we consider the monogenean fauna of the invasive fishes detected in our study to be potential co-invaders until more extensive surveys of wild populations prove otherwise.

**Table 8:** List of invasive Indian freshwater ornamental fish.

Fish species	Key references
*Barbonymus altus*	[83]
*Carassius auratus*	[80]
*Cyprinus carpio*	[91]
*Osphronemus goramy*	[53, 77, 78]
Piaractus brachypomus	[96]
*Poecilia reticulata*	[11]
*Pterygoplichthys disjunctivus*	[12, 93]
*Trichopodus trichopterus*	[53]
*Xiphophorus helleri*	[50, 81]
*Xiphophorus maculatus*	[78]

#### Additional parasites

Although the parasite sampling procedures were designed principally to detect monogenean parasites, we isolated several other metazoan parasites as well. For example, we found crustacean parasites from *C*. *auratus*. These included one copepod parasite species (*Lernaea cyprinacea* Linnaeus, 1758) and three brachyuran parasite species (*Argulus coregoni* Thorell, 1866, *Argulus japonicus* Thiele, 1899, and *Argulus foliaceus* Linnaeus, 1758). We also found some trematodes, cestodes, and nematodes but ignored them due to a lack of taxonomic expertise in these taxa. Nevertheless, given the risk of translocating parasites within these taxa between continents with uncritical ornamental fish trade [18, 64], future studies should also meticulously examine trematodes, cestodes, nematodes, crustaceans, acanthocephalans, and myxozoans. Monogeneans clearly have pathogenic potential, and because they have a single-host life cycle, they may propagate fast and colonise new hosts in new geographic regions. This, however, may not justify the exclusion of other helminth types from the regular parasitological monitoring of ornamental fish.

#### Illegal trafficking of ornamental fish and their parasites

Illegal wildlife trade presents many threats to public health, wildlife, and the ecosystem, serving as a primary driver of emerging infectious diseases [49]. The impact of introduced monogenean species on the native fish species is well-documented. These types of impacts have been associated with capsalid *Nitzschia sturionis* (Abildgaard, 1794) Krøyer, 1852 (Capsalidae), translocated from the Caspian Sea on *Acipenser nudiventris* Lovetsky, 1828 (Acipenseriformes: Acipenseridae) to the Aral Sea [29]; gyrodactylid *Gyrodactylus salaris* Malmberg, 1957 from Sweden on *Salmo salar* Linnaeus, 1758 (Salmoniformes: Salmonidae) to Norway [47]; and a diclidophorid *Neoheterobothrium hirame* Ogawa, 1999 from North America on *Paralichthys olivaceus* Temminck and Schlegel, 1846 (Pleuronectiformes: Paralichthyidae) to Japan [5].

Of the 27 fish species sampled, 11 (41%) were not on the government of India’s indicative list, meaning they were brought into the country through illegal trafficking. These fish are *B*. *altus*, *C*. *socolofi*, *G*. *ternetzi*, *H*. *temminckii*, *H*. *erythrozonus*, *M*. *hypsauchen*, *M*. *ramirezi*, *P*. *brachypomus, P*. *sphenops*, *P*. *disjunctivus*, and *S*. *argus*. Fish that have been illegally smuggled into the country present an especially great hazard because they have bypassed the import risk analysis and quarantine procedures of the importing country, and therefore, have a high probability of carrying exotic parasites. This is justified by the fact that eight of 11 (72.72%) illegally imported fishes were found to be infected with monogenean parasites, compared to nine of the 16 (56.25%) that are available on the indicative list and had passed through the quarantine process. Such illegal imports, therefore, may accelerate parasite spread, leading to disease outbreaks. As such, these fish should be added to the “indicative list” and placed in quarantine on arrival, or their sale should be considered a criminal act, comparable to wildlife smuggling, and declared punishable by severe penalties.

#### Risks to importing countries, especially India, with the changing global climate

Host-parasite interactions may be influenced by environmental conditions, including climate change [65], which can directly affect parasite prevalence and intensity or indirectly impact the phenology of both hosts and parasites [62]. However, predicting the consequences of climate change on host-parasite dynamics is difficult due to limited or conflicting empirical evidence available. Nonetheless, it is widely accepted that global climate change will influence the distribution of parasite diseases [3]. This could facilitate the introduction of parasites into the new environments, aggravating the already well-documented devastating consequences on endemic teleost populations following the uncritical import and release of fish [31] and their parasites [18] into natural waters.

The survival and reproduction of various parasites depend on a range of climate-related abiotic factors, such as salinity, pH, humidity, and, most notably, temperature [111]. Higher temperatures, for example, often increase parasite growth, reproduction, and infectivity [61, 92]. Therefore, fluctuations in these basic ecological factors in regions where exotic parasites have been introduced may benefit both endemic and introduced parasite species as climate conditions change. Of special note, in this context, the reproductive rates of both oviparous [15] and viviparous monogeneans [36, 56, 102] are positively correlated to temperature. Although climate change is a global concern, India is particularly vulnerable to its impacts due to its fast population growth and the general inability of its infrastructure and public services to adapt to anticipated adverse effects [86].

## Conclusions and call for action

Understanding the spectrum of exotic parasites and pathogens, particularly under changing ecological and environmental conditions of intercontinental translocations, is crucial in determining the associated risks and appropriate management actions. Our study, which is the first large-scale quantitative parasitological investigation of a wide range of exotic ornamental fishes in India, established the abundance and diversity of monogenean parasites in freshwater ornamental fishes imported into the country. Morpho-molecular profiling revealed the presence of monogenean parasites in 17 of the 27 exotic ornamental fish examined. Twenty-six monogenean species were confirmed as co-introduced, nearly half of which were considered potential co-invaders. The prevalence and intensity of infection were generally high for most of the species studied, which can only be attributable to the inadequate risk assessment, detection, diagnosis, and management of quarantine systems at Indian borders.

Two key points are made in concluding this study. First, the ornamental trade is a strong driver of the transboundary translocation of monogenean parasites. Second, current biosecurity measures designed to prevent the introduction of exotic parasites into India are less stringent than previously thought, requiring alternative approaches to be explored or existing protocols to be upgraded. We hypothesise that these fishes may have similarly introduced several other protistan and metazoan parasites into India. This possibility should be investigated by taxonomists who specialize in these groups.

The data generated in this study serve three important purposes. First, the data demonstrate the efficiency of the ornamental fish trade in transporting monogenean parasites across continents. Second, the data represent essential baseline information for the prevention and management of exotic monogenean parasites in Indian aquaria and ponds. Third, and most importantly, the data raise concerns about the vulnerability of India’s quarantine barrier to the infiltration of monogenean parasites (and, potentially, other parasites).

A detailed review of the efforts made by the Government of India to protect native biodiversity from exotic species is beyond the scope of this paper. Still, it can be said that the Government of India is increasingly aware of the serious consequences of introducing exotic animals, including ornamental fishes, and their parasites. As a result, various policies have been enacted to prevent and control them. Specifically, in 2015, the Ministry of Agriculture and Farmers Welfare implemented “Guidelines for the Import of Ornamental Fishes into India” to regulate the introduction of exotic aquatic ornamental animals. In 2017, the ministry replaced these guidelines with the “Sanitary Protocol for Import of ornamental fishes into India” (see Introduction). The new “protocol” prioritises prevention of disease introduction and ensuring the health and safety of humans and the Indian aquatic environment.

Most recently, the Wildlife Protection Act of 1972, amended in 2022, added section 62A. (1), which states that “The Central Government may, by notification, regulate or prohibit the import, trade, possession or proliferation of invasive alien species which pose a threat to the wild life or habitat in India”. In 2024, the Ministry of Environment, Forest, and Climate Change updated its National Biodiversity Strategy and Action Plan, a framework used by countries to implement the Convention on Biological Diversity at the national level, establishing 23 National Biodiversity Targets, with Target 6 focussing specifically on the management of invasive alien species. Despite these seemingly strong provisions, there are clear limitations (to which this paper is a testimony); some of these were reviewed by Tripathi [99].

In this context, based on the results, we offer the following recommendations for reducing the co-transport and release of potentially harmful fish parasite species:

1. Separate risk analysis and quarantine provisions should be implemented for fish as pets and for the parasites they may carry.
2. Quarantine facilities should include highly specialised diagnostic laboratories employing a wide range of registered fish health professionals, including particularly parasite taxonomists.
3. Molecular-genetic identification, based on conventional and real-time PCR along with Sanger sequencing, Illumina sequencing, or their combination should be used in addition to traditional methods of identification (light microscopy) to ensure the rapid, accurate, and reliable detection and identification of parasites, especially monogeneans.
4. A continuous and mandatory surveillance and reporting system should be developed and enforced.
5. A dedicated “Central Institute of Invasive Species Management” should be established as a multidisciplinary research, teaching, and extension unit to coordinate the management of invasive species throughout India.
6. A dedicated and sustained funding mechanism should be developed to allow for fast responses to and the effective management of exotic fish parasites.
